# Parallels in Amphibian and Bat Declines from Pathogenic Fungi

**DOI:** 10.3201/eid1093.120707

**Published:** 2013-03

**Authors:** Evan A. Eskew, Brian D. Todd

**Affiliations:** Author affiliation: University of California, Davis, California, USA

**Keywords:** adaptive immunity, amphibians, Ascomycota, bats, biodiversity, Chytridiomycota, host-pathogen interactions, immunocompromised hosts, immunology, infectious disease outbreak, infectious disease reservoirs, infectious disease transmission, innate immunity, pathogenicity factors, species extinction, virulence factors, fungi

## Abstract

Pathogenic fungi have substantial effects on global biodiversity, and 2 emerging pathogenic species—the chytridiomycete *Batrachochytrium dendrobatidis*, which causes chytridiomycosis in amphibians, and the ascomycete *Geomyces destructans*, which causes white-nose syndrome in hibernating bats—are implicated in the widespread decline of their vertebrate hosts. We synthesized current knowledge for chytridiomycosis and white-nose syndrome regarding disease emergence, environmental reservoirs, life history characteristics of the host, and host–pathogen interactions. We found striking similarities between these aspects of chytridiomycosis and white-nose syndrome, and the research that we review and propose should help guide management of future emerging fungal diseases.

Fungi and fungus-like organisms have been recognized historically as prominent plant pathogens that can have detrimental effects on agricultural crops and wild flora (*1*,*2*). Fisher et al. (*3*) recently reviewed the increasing role and recognition of pathogenic fungi that affect global biodiversity. Their analyses showed that most (91%) recent extinctions and extirpations caused by fungal disease have affected animals rather than plants. In particular, 2 emerging pathogenic fungi—the chytridiomycete *Batrachochytrium dendrobatidis*, which causes chytridiomycosis in amphibians (*4*,*5*), and the ascomycete *Geomyces destructans*, which causes white-nose syndrome (WNS) in hibernating bats (*6*)—are implicated in the widespread decline of their vertebrate hosts. In general, increased global biosecurity and monitoring are recommended to prevent and manage emerging fungal diseases (*3*), but there are also pressing research needs that can help specifically address these 2 devastating pathogenic fungi. We call attention to parallels between chytridiomycosis and WNS and highlight areas where urgent research is required (Table). Comparison of these diseases also illustrates broader themes and questions that can be used to direct research on future emerging fungal diseases.

**Table:** Current knowledge and unresolved research questions regarding the pathogenic fungi *Batrachochytrium dendrobatidis* and *Geomyces destructans*, the causative agents of chytridiomycosis and WNS, respectively*

Area of knowledge	*B. dendrobatidis*			*G. destructans*	
	Current knowledge	Unresolved research questions		Current knowledge	Unresolved research questions
Disease emergence	Multiple regions of endemism and 1 widely introduced hypervirulent lineage (*7–9*)	How and from where did the hypervirulent lineage emerge?		Limited genetic differentiation in North America (*10*)	How do strains from North America and Europe compare genetically, and is genetic variation greater in Europe, suggesting historic endemism?
				Possibly endemic to Europe and introduced to North America (*6**,**11**,**12*)	
Abiotic reservoirs	Can survive in water and soil (*13,14*)	Can* B. dendrobatidis* form desiccation-resistant resting spores? Can *B. dendrobatidis* survive and reproduce as a saprophytic, nonparasitic form?		Apparent persistence in soils and on cave walls (*12**,**15*)	How widespread is* G. destructans* in the environment? Can *G. destructans* survive and reproduce as a saprophytic, nonparasitic form?
Biotic reservoirs	Host generalist pathogen of amphibians (*4**,**5*)	Can* B. dendrobatidis* complete its life cycle on other vertebrate hosts?		Host generalist pathogen of bats (*6*)	Can* G. destructans* infect or persist on other vertebrates?
	Can also infect reptiles, nematodes, and waterfowl (*16**–**18*)				
Life history and infection risk of the host	Aquatic, biphasic, tropical amphibian species at greatest risk for chytridiomycosis (*19*)	To what extent can life history characteristics of the host predict global patterns of disease-related population decline among amphibian species?		Bat species that hibernate experience most deaths from WNS (*20*)	Are only those species that hibernate susceptible to population decline from WNS? What role does life history of the host play in predicting species declines and extinctions from WNS?
Host–pathogen interactions	Antimicrobial peptides and antifungal metabolites from skin-associated bacteria contribute to* B. dendrobatidis* resistance (*21*)	What is the immune response of* B. dendrobatidis*–tolerant hosts to infection?		Host immune down-regulation during hibernation probably important to WNS progression (*24*)	What is the host immune response to* G. destructans* infection?
	Susceptible species appear to show little innate or adaptive immune response to* B. dendrobatidis* infection (*22,23*)	Does *B. dendrobatidis* evade the amphibian immune system through activity of secreted proteases?			How does host immunity vary seasonally? What role does immune function play in the observed winter season/hibernation mortality from WNS? Do proteases contribute to pathogenicity of *G. destructans*?

*WNS, white-nose syndrome.

## Disease Emergence

Effective control of any disease requires understanding of the processes that have resulted in its emergence. The study of a pathogen’s geographic origin and phylogenetic history often provides critical insight in this regard. In general, infectious diseases can emerge from 2 distinct scenarios: 1) the geographic spread of a novel pathogen into a new area with naive hosts or 2) a shift in pathogenicity or host specificity of an endemic pathogen as a result of environmental changes that alter host–pathogen interactions (*25*). Current evidence predominantly suggests that chytridiomycosis and WNS outbreaks are driven by anthropogenic transport of novel pathogenic fungi into new geographic regions.

As chytridiomycosis began emerging globally, it was initially unclear whether *B. dendrobatidis* was newly introduced in regions affected by chytridiomycosis or whether it was globally endemic and had recently increased in pathogenicity as a result of environmental degradation or climate change (*4*,*5*,*25*). Spatiotemporal patterns of declines in amphibian populations from chytridiomycosis and population genetics data on *B. dendrobatidis* provide increasing evidence that the pathogen has been recently introduced in many areas (*4*,*5*). Wave-like patterns of amphibian deaths from chytridiomycosis have been documented at multiple sites globally, and these spatiotemporal dynamics suggest that *B. dendrobatidis* behaves as a novel pathogen spreading geographically through naive host populations (*4*). In addition, *B. dendrobatidis* strains isolated from across the world have low levels of genetic diversity, consistent with a pathogen that has recently undergone rapid range expansion (*4*,*5*).

In early research, Africa was proposed as the place of origin for *B. dendrobatidis*, and the African clawed frog (*Xenopus laevis*), which is not susceptible to chytridiomycosis, was thought to be a primary carrier responsible for global dispersal of *B. dendrobatidis* out of Africa (*26*). This amphibian species was used in human pregnancy assays beginning in the mid-1930s and later became a widely used biological model. On the basis of museum evidence that some African clawed frogs were infected with *B. dendrobatidis* in the late 1930s, Weldon et al. (*26*) hypothesized that global demand for the species resulted in the spread of *B. dendrobatidis* out of Africa to other areas where it subsequently became established in native, naive wild populations. Amphibian species in pet stores, zoos, museums, and the food market are now known to carry *B. dendrobatidis* infections (*4*,*7*,*8*), and the international trade of amphibians is thought to be a major factor driving the spread of* B. dendrobatidis*, with other key carrier species, such as the North American bullfrog (*Lithobates catesbeianus*), contributing to the pathogen’s global invasion (*4*,*5*,*9*,*26*,*27*).

The leading hypothesis, based on population genomic evidence, posits that a single hypervirulent *B. dendrobatidis* lineage is largely responsible for global chytridiomycosis-related amphibian declines and that the emergence of this lineage likely resulted from anthropogenic pathogen transport (*9*). Anthropogenic activity might have helped generate this hypervirulent strain by promoting fungal lineage mixing. Lineage mixing enables distantly related pathogen strains to outbreed, and this process can result in the emergence of novel, virulent genotypes that spread rapidly through susceptible host populations, as appears to have occurred with chytridiomycosis (*9*).

Studies of population genetics have contributed substantially to our understanding of *B. dendrobatidis* as a novel pathogen in regions affected by chytridiomycosis; however, wider global sampling and phylogenetic analysis of the fungus is needed (Table). Africa remains a critical region for the study of endemic *B. dendrobatidis* diversity, but research on strains isolated worldwide suggests that other strains also are endemic to Eurasia (*7*–*9*). Although these endemic strains might play a relatively small role in the global decline of amphibian populations, further study and broader sampling of *B. dendrobatidis* can improve our understanding of its phylogenetic history and shed light on the possible contributions of lineage mixing and genetic variation to pathogenicity.

Our ability to accurately reconstruct* B. dendrobatidis* phylogeny is hampered by the limited number of* B. dendrobatidis* samples collected from species other than those susceptible to chytridiomycosis. As a result, additional cryptic, endemic lineages may remain overlooked or undersampled because they are associated with* B. dendrobatidis*–tolerant hosts (*7*,*8*), and environmental reservoirs could represent another source of undiscovered pathogen diversity. The global distribution of* B. dendrobatidis* before chytridiomycosis emergence and the geographic origin of the hypervirulent lineage remain uncertain (*27*), but both African (*26*) and Japanese (*7*) amphibian species appear to have been asymptomatic* B. dendrobatidis* hosts decades before amphibian declines were reported in other regions.

The origin of *G. destructans* and processes resulting in WNS emergence are unresolved. Bats in Europe are known to carry* G. destructans* asymptomatically, which has led to speculation that *G. destructans* was introduced from the Old World to susceptible North American bats, probably through human transport (*6*,*11*). Genetic markers show no differentiation among North American* G. destructans* samples collected across the expanding area of WNS, suggesting that* G. destructans* was recently introduced and is spreading rapidly through North America (*10*). Furthermore, the first observation of WNS in North America occurred in a cave that had high levels of tourism, providing circumstantial evidence for a link between human activity and* G. destructans* introduction (*11*). Although no global population genomic study has been conducted for* G. destructans*, the possibility that lineage mixing and recombination among fungal strains has contributed to WNS outbreaks is intriguing. In a laboratory study, North American bats died when infected with North American or European strains of* G. destructans* (*28*). These results were interpreted as further indirect evidence that* G. destructans* is novel to North America. However, recent sampling suggests that bat hibernacula host a diversity of previously uncharacterized *Geomyces* spp (*15*), and given our limited understanding of the global distribution of* G. destructans* strains and their phylogenetic relationships (*6*), it remains possible that* G. destructans* is endemic to North America. The distribution of* G. destructans* lineages in North America, similar to *B. dendrobatidis* distribution in some parts of Asia (*7*,*8*), might therefore include endemic, avirulent fungal strains in addition to recently introduced, potentially more virulent lineages. Currently, however, the lack of evidence for* G. destructans* presence in North America outside of WNS-affected areas supports the hypothesis that the fungus is indeed novel to this region (*6*). As with *B. dendrobatidis*, firm conclusions regarding the geographic origins of* G. destructans* await a much broader global sampling of* G. destructans* combined with fine-scale population genetic analyses of highly polymorphic markers (Table) (*6*).

## Environmental Reservoirs

Theoretical work suggests that if environmental reservoirs (abiotic substrates or alternative biotic hosts that enable pathogen persistence) exist, diseases can have serious population-level effects on host species and even precipitate host extinction (*29*). The existence of multiple hosts can buffer a pathogen against population fluctuations in focal host species, and tolerant species may therefore increase pathogen burden for susceptible species by supporting high pathogen densities. Abiotic reservoirs also enable pathogen persistence when suitable vertebrate hosts are rare or absent, which also may increase pathogen burden for susceptible species. For example, mathematical models show that the risk for host extinction from chytridiomycosis increases when fungal zoospores have long residence time in the environment or if* B. dendrobatidis* can reproduce apart from amphibian hosts (*30*). Because of their effects on disease dynamics, recognition of the full range of environmental reservoirs is essential for effective management of any emerging disease.

There is strong reason to suspect that* B. dendrobatidis* and* G. destructans* have environmental reservoirs. In general, fungi may be unique pathogens because many can persist in the environment apart from animal hosts, yet environmental pressures can select for fungal traits that contribute to virulence during infection of certain host organisms (*31*). Fungi in the phylum Chytridiomycota have a nearly global distribution and occupy roles as heterotrophs and saprobes in water and soil (*32*). Similarly, multiple species of *Geomyces* are saprophytic (*20*). Given the ecologic characteristics of their broader taxonomic groups, it is perhaps unsurprising that environmental sampling is now revealing a variety of reservoirs for* B. dendrobatidis* and* G. destructans*.

Further work is needed to fully characterize the abiotic reservoirs of both pathogens (Table). As an aquatic fungus, *B. dendrobatidis* can survive in various aqueous media for several weeks (*13*) and may grow in moist soil (*14*). Although these laboratory studies demonstrate the range of conditions under which *B. dendrobatidis* might occur, patterns of fungal persistence in natural environments are yet to be studied intensively. In addition, Di Rosa et al. (*33*) have suggested that *B. dendrobatidis* may exist as a stress-tolerant resting spore, representing another abiotic reservoir for the fungus. However, whether the structure in question is in fact an alternate life stage of* B. dendrobatidis* remains unclear (*5*). Environmental distribution of* G. destructans* also is poorly understood, but the pathogen has been detected in soil samples (*15*), and viable spores have been collected from cave walls in bat hibernacula (*12*), both factors that suggest the fungus has abiotic reservoirs.

Chytridiomycosis and WNS are caused by host-generalist pathogens that have multiple biotic reservoirs. *B. dendrobatidis* infects hundreds of amphibian species globally (*4*,*5*). In North America, WNS has spread among 9 bat species (*6*). Although research has predominately focused on the prevalence of* B. dendrobatidis* and* G. destructans* in taxa negatively affected by those pathogens (amphibians and bats, respectively), wider sampling of these fungi is warranted (Table). For example, despite more than a decade of* B. dendrobatidis*–related research, it was only recently discovered that the fungus may infect reptiles without causing disease (*16*). Furthermore, experimental* B. dendrobatidis* infections can lead to death of nematodes, although whether these organisms frequently serve as hosts for* B. dendrobatidis* in the wild is unclear (*17*). *B. dendrobatidis* has also recently been found on migratory waterfowl and may be carried among bodies of water by the infected keratinized feet and webbings of such birds (*18*). Whether bats are the only vertebrates capable of hosting* G. destructans* remains to be seen. Raccoons, bears, rodents, or other mammals that frequent cave systems where* G. destructans* is present are the most likely candidates to serve as alternative hosts (Table). Mammalian species that experience lowered body temperatures during winter torpor or hibernation may warrant particular attention, given* G. destructans*’s preference for psychrophilic growth (*6*,*34*).

## Life History Characteristics and Infection Risk of the Host

Host life history characteristics can play a critical role in determining infection risk, and some amphibians and bats appear to be susceptible to pathogenic fungi in part because of their life histories. Amphibians often reach high population densities and have increased contact rates when breeding at aquatic sites; both factors promote pathogen transmission (*35*). Amphibians also may have intraspecific host reservoirs because of their complex life histories (i.e., aquatic larvae experience persistent sublethal infections and expose terrestrial adults that visit aquatic sites), enabling pathogen persistence within amphibian populations (*35*). Many amphibians undergo immune suppression during metamorphosis, increasing their infection risk during this life stage (*35*). These factors probably explain, in part, the results of a study from Central America that found amphibian species that were highly aquatic were those most likely to suffer* B. dendrobatidis*–related population declines or extirpations (*19*). Analyses of amphibian communities in other regions are needed to determine whether life history characteristics of the host are broadly useful in predicting global chytridiomycosis-associated declines in amphibian populations (Table).

Just as some amphibian species are particularly susceptible to chytridiomycosis because of their life histories, the species of North American bats that are affected by WNS have life history characteristics that similarly predispose them to disease outbreaks. Bat populations in North America that are declining from WNS are known to breed, roost, and hibernate communally in large aggregations in cave systems where increased densities and contact between bats can facilitate pathogen transmission (*6*,*20*). There is speculation that if host density during hibernation influences WNS severity, this life history attribute may help explain the lack of WNS in Europe, where bat species typically form smaller hibernation aggregations than in North America (*11*). Bats in warm Mediterranean regions of Europe that have short hibernation periods appear to be free of* G. destructans* (*12*), which further supports a role for host life history in mediating pathogen transmission. In addition, the down-regulation of immune function in bats during hibernation (*24*) is roughly analogous to the immune suppression that occurs during amphibian metamorphosis, a trait that seems to be linked to increased infection susceptibility (*35*). WNS emerged in the northeastern United States but is rapidly spreading southwestward toward regions of high bat species richness where many species do not hibernate. Whether those species in the southwestern United States that do not hibernate will be at risk for WNS is unclear (*20*). To best predict infection risk and effectively manage amphibian and bat diversity, the degree to which life history and ecologic variation among hosts influence disease susceptibility needs to be better understood (Table). In this regard, broad epidemiologic studies and analyses of incoming reports and publications could yield key insights.

## Host–Pathogen Interactions

The pathologies of chytridiomycosis and WNS are broadly similar in that both diseases result from infection by dermatophyte fungi that can severely disrupt host physiology (*5*,*6*,*24*,*27*). Amphibian skin plays a major role in physiologic regulation, and* B. dendrobatidis* infection of this tissue causes electrolyte imbalance in host individuals that ultimately leads to death from cardiac arrest (*27*). In bats, WNS results in death by increasing arousal frequency during hibernation, which depletes bat energy reserves (*28*). These behavioral changes in bats may ultimately be a reaction to the disruption of physiologic processes, including water balance, gas exchange, and thermoregulation caused by* G. destructans* infection of the wing membrane structure (*24*). However, in amphibians and bats, considerable interspecific variation exists in disease-related illness and death from these fungal infections (*4*–*6*). One unresolved question for both chytridiomycosis and WNS is the degree to which interspecific differences in host–pathogen interactions underlie variation in disease outcome.

Some evidence indicates that host defenses mediate species-specific responses to chytridiomycosis and WNS. In amphibians, basic physiologic and anatomic traits that vary among species, such as skin sloughing rate and skin thickness, could provide innate defenses against the keratinophilic activity of* B. dendrobatidis* (*5*,*27*). Research also has shown that species-specific assemblages of skin-associated bacteria and suites of antimicrobial peptides affect amphibian susceptibility to chytridiomycosis (*21*). Not surprisingly, innate and adaptive amphibian immune function probably plays a critical role in determining chytridiomycosis disease outcome (*27*). The few studies that have investigated host immune response to chytridiomycosis suggest that susceptible frog species have a weak adaptive immune response to* B. dendrobatidis* infection (*22*,*23*). In contrast, work on the disease-resistant frog *Xenopus laevis* suggests that innate and adaptive immune components constitute host response to* B. dendrobatidis* infection in this species (*36*). Examining host immune response to* B. dendrobatidis* among closely related amphibians that differ in disease susceptibility would be of tremendous value to more fully elucidate the immune genes, pathways, and responses that contribute to* B. dendrobatidis* tolerance (Table). Bat immune response to* G. destructans* infection is poorly characterized (*6*), but species may be susceptible to WNS partly because of natural immune system down-regulation during hibernation (*24*). This current gap in knowledge deserves particular attention given that differences in host immune function between bat species in North America and Europe could account for their differential susceptibility to WNS (*6*,*11*). Immunogenetic studies are urgently needed to better understand the immunologic mechanisms driving WNS-associated population declines among bat species in North America. Comparisons between susceptible and nonsusceptible species, species in North America and Europe, and active and hibernating bats will be particularly important in this regard (Table).

Alternatively, disease processes in chytridiomycosis and WNS may largely reflect activity of the pathogenic fungi themselves, and further work is needed to understand the factors that drive pathogenicity in these fungal species (Table). Researchers have hypothesized that* B. dendrobatidis* may evade or suppress the amphibian immune system (*21*,*22*), but understanding of molecular and cellular mechanisms for such activity is lacking. Recent genomic comparisons with other fungi suggest that expansions of protease gene families during the recent evolutionary history of *B. dendrobatidis* might account for the pathogen’s ability to colonize amphibian skin and evade the amphibian immune system (*37*). Because secreted proteases also appear to contribute to virulence in other dermatophyte fungi (*27*), activity of these genes could help explain pathogenicity of* G. destructans* as well. *G. destructans* is known to produce secretory proteases in culture (*34*), although the role of these proteins during pathogenesis of WNS has yet to be elucidated.

## Conclusions

In general, the fungal diseases chytridiomycosis and WNS show striking similarities. Both diseases appear to be driven by novel pathogens introduced to new geographic regions by human transport. *B. dendrobatidis* and* G. destructans* are host-generalist pathogens with abiotic reservoirs that can persist even when the density of host species is low. The life history characteristics of many amphibians and bats result in high host densities, high rates of host contact with the fungi, and depressed immune function during specific life stages or seasons, all of which are factors that contribute to pathogen persistence and transmission within a host community. Finally, both* B. dendrobatidis* and* G. destructans* are dermatophyte fungi that appear to successfully overcome host defenses in some of their primary host species.

Recognizing the commonalities between chytridiomycosis and WNS can help identify management efforts that may best address future emerging fungal diseases. Because anthropogenic transport of novel pathogens appears to play a major role in fungal disease emergence, increased global biosecurity aimed at minimizing the spread of invasive pathogens may be among the most effective fungal disease mitigation strategies (*3*). Although biosecurity will aid in detecting and preventing transport of many pathogen types, these efforts are particularly critical for controlling pathogenic fungi, given that they may infect a broad range of host species and persist on abiotic substrates long enough to increase their likelihood of successful dispersal to new areas. In addition, disease monitoring might focus on animals with life history characteristics that increase their risk for pathogen exposure or illness following pathogen introduction into a population. Species that exhibit social behavior or other intraspecific interactions might be especially vulnerable to disease outbreaks. For example, an opportunistic pathogenic fungus was recently identified in a declining population of timber rattlesnakes (*38*), and susceptibility to outbreaks of fungal disease might be predicted for this and similar species given their communal denning behavior and potential for lowered immune function during hibernation.

Although pathogen virulence and disease outbreaks of are extremely difficult to predict, scientists focusing on wildlife disease might prioritize research on fungi closely related to currently pathogenic species while remaining vigilant of fungal disease outbreaks in new host species. Pathogenic fungi have primarily been viewed as threats to ectothermic organisms because ectotherms can have low body temperatures that are suitable for the growth of many fungi (*31*,*39*). *B. dendrobatidis* (*4*,*5*) and* G. destructans* (*6*,*20*) are psychrophilic species with growth optima at lower temperatures (17°–25°C for* B. dendrobatidis*, 10°–15°C for* G. destructans*). In contrast, endothermic body temperatures generally exceed the upper thermal tolerance limits of fungi, a factor that might partially explain why WNS affects bats during hibernation when their internal temperature is lowered and immune function is inhibited (*24*,*39*). However, some researchers hypothesize that anthropogenic climate warming will select for increasing heat tolerance in fungi, presumably resulting in a greater number of fungi capable of surviving in the range of endothermic body temperatures (*40*). Regional warming and associated changes in fungal heat tolerance may therefore create new pathogen risks for endothermic vertebrate hosts. In conclusion, we suggest psychrophilic fungal species probably will continue to be major pathogens of ectothermic vertebrates, whereas fungi with thermal tolerances closer to endothermic body temperatures may represent increasing threats to endotherms. Despite the associated challenges, the recent devastating effects of chytridiomycosis and WNS suggest that biologists and epidemiologists should give greater attention to pathogenic fungi if they wish to preserve vertebrate biodiversity in a rapidly changing global environment.

## References

[1] Anderson PK, Cunningham AA, Patel NG, Morales FJ, Epstein PR, Daszak P. Emerging infectious diseases of plants: pathogen pollution, climate change and agrotechnology drivers. Trends Ecol Evol. 2004;19:535–44. 10.1016/j.tree.2004.07.021 16701319

[2] Desprez-Loustau M-L, Robin C, Buée M, Courtecuisse R, Garbaye J, Suffert F, The fungal dimension of biological invasions. Trends Ecol Evol. 2007;22:472–80. 10.1016/j.tree.2007.04.005 17509727

[3] Fisher MC, Henk DA, Briggs CJ, Brownstein JS, Madoff LC, McCraw SL, Emerging fungal threats to animal, plant and ecosystem health. Nature. 2012;484:186–94. 10.1038/nature10947 22498624 PMC3821985

[4] Fisher MC, Garner TWJ, Walker SF. Global emergence of *Batrachochytrium dendrobatidis* and amphibian chytridiomycosis in space, time, and host. Annu Rev Microbiol. 2009;63:291–310 . 10.1146/annurev.micro.091208.073435 19575560

[5] Kilpatrick AM, Briggs CJ, Daszak P. The ecology and impact of chytridiomycosis: an emerging disease of amphibians. Trends Ecol Evol. 2010;25:109–18. 10.1016/j.tree.2009.07.011 19836101

[6] Puechmaille SJ, Frick WF, Kunz TH, Racey PA, Voigt CC, Wibbelt G, White-nose syndrome: is this emerging disease a threat to European bats? Trends Ecol Evol. 2011;26:570–6 . 10.1016/j.tree.2011.06.013 21835492

[7] Goka K, Yokoyama J, Une Y, Kuroki T, Suzuki K, Nakahara M, Amphibian chytridiomycosis in Japan: distribution, haplotypes and possible route of entry into Japan. Mol Ecol. 2009;18:4757–74. 10.1111/j.1365-294X.2009.04384.x 19840263

[8] Bai C, Liu X, Fisher MC, Garner TWJ, Li Y. Global and endemic Asian lineages of the emerging pathogenic fungus *Batrachochytrium dendrobatidis* widely infect amphibians in China. Divers Distrib. 2012;18:307–18. 10.1111/j.1472-4642.2011.00878.x

[9] Farrer RA, Weinert LA, Bielby J, Garner TWJ, Balloux F, Clare F, Multiple emergences of genetically diverse amphibian-infecting chytrids include a globalized hypervirulent recombinant lineage. Proc Natl Acad Sci U S A. 2011;108:18732–6. 10.1073/pnas.1111915108 22065772 PMC3219125

[10] Ren P, Haman KH, Last LA, Rajkumar SS, Keel MK, Chaturvedi V. Clonal spread of *Geoymces destructans* among bats, midwestern and southern United States. Emerg Infect Dis. 2012;18:883–5. 10.3201/eid1805.111711 22516471 PMC3358064

[11] Wibbelt G, Kurth A, Hellmann D, Weishaar M, Barlow A, Veith M, White-nose syndrome fungus (*Geomyces destructans*) in bats, Europe. Emerg Infect Dis. 2010;16:1237–43. 10.3201/eid1608.100002 20678317 PMC3298319

[12] Puechmaille SJ, Wibbelt G, Korn V, Fuller H, Forget F, Mühldorfer K, Pan-European distribution of white-nose syndrome fungus (*Geomyces destructans*) not associated with mass mortality. PLoS ONE. 2011;6:e19167. 10.1371/journal.pone.0019167 21556356 PMC3083413

[13] Johnson ML, Speare R. Survival of *Batrachochytrium dendrobatidis* in water: quarantine and disease control implications. Emerg Infect Dis. 2003;9:922–5. 10.3201/eid0908.030145 12967488 PMC3020615

[14] Johnson ML, Speare R. Possible modes of dissemination of the amphibian chytrid *Batrachochytrium dendrobatidis* in the environment. Dis Aquat Organ. 2005;65:181–6. 10.3354/dao065181 16119886

[15] Lindner DL, Gargas A, Lorch JM, Banik MT, Glaeser J, Kunz TH, DNA-based detection of the fungal pathogen *Geomyces destructans* in soils from bat hibernacula. Mycologia. 2011;103:241–6. 10.3852/10-262 20952799

[16] Kilburn VL, Ibáñez R, Green DM. Reptiles as potential vectors and hosts of the amphibian pathogen *Batrachochytrium dendrobatidis* in Panama. Dis Aquat Organ. 2011;97:127–34. 10.3354/dao02409 22303629

[17] Shapard EJ, Moss AS, San Francisco MJ. *Batrachochytrium dendrobatidis* can infect and cause mortality in the nematode *Caenorhabditis elegans.* Mycopathologia. 2012;173:121–6. 10.1007/s11046-011-9470-2 22002554

[18] Garmyn A, Van Rooij P, Pasmans F, Hellebuyck T, Van Den Broeck W, Haesebrouck F, Waterfowl: potential environmental reservoirs of the chytrid fungus *Batrachochytrium dendrobatidis.* PLoS ONE. 2012;7:e35038. 10.1371/journal.pone.0035038 22514705 PMC3325947

[19] Lips KR, Reeve JD, Witters LR. Ecological traits predicting amphibian population declines in Central America. Conserv Biol. 2003;17:1078–88. 10.1046/j.1523-1739.2003.01623.x

[20] Foley J, Clifford D, Castle K, Cryan P, Ostfeld RS. Investigating and managing the rapid emergence of white-nose syndrome, a novel, fatal, infectious disease of hibernating bats. Conserv Biol. 2011;25:223–31 . 21284732 10.1111/j.1523-1739.2010.01638.x

[21] Rollins-Smith LA, Ramsey JP, Pask JD, Reinert LK, Woodhams DC. Amphibian immune defenses against chytridiomycosis: impacts of changing environments. Integr Comp Biol. 2011;51:552–62. 10.1093/icb/icr095 21816807

[22] Rosenblum EB, Poorten TJ, Settles M, Murdoch GK. Only skin deep: shared genetic response to the deadly chytrid fungus in susceptible frog species. Mol Ecol. 2012;21:3110–20. 10.1111/j.1365-294X.2012.05481.x 22332717

[23] Ribas L, Li M-S, Doddington BJ, Robert J, Seidel JA, Kroll JS, Expression profiling the temperature-dependent amphibian response to infection by *Batrachochytrium dendrobatidis.* PLoS ONE. 2009;4:e8408. 10.1371/journal.pone.0008408 20027316 PMC2794374

[24] Cryan PM, Meteyer CU, Boyles JG, Blehert DS. Wing pathology of white-nose syndrome in bats suggests life-threatening disruption of physiology. BMC Biol. 2010;8:135. 10.1186/1741-7007-8-135 21070683 PMC2984388

[25] Rachowicz LJ, Hero J-M, Alford RA, Taylor JW, Morgan JAT, Vredenburg VT, The novel and endemic pathogen hypotheses: competing explanations for the origin of emerging infectious diseases of wildlife. Conserv Biol. 2005;19:1441–8. 10.1111/j.1523-1739.2005.00255.x

[26] Weldon C, du Preez LH, Hyatt AD, Muller R, Speare R. Origin of the amphibian chytrid fungus. Emerg Infect Dis. 2004;10:2100–5. 10.3201/eid1012.030804 15663845 PMC3323396

[27] Fisher MC, Farrer RA. Outbreaks and the emergence of novel fungal infections: lessons from the panzootic of amphibian chytridiomycosis. The Journal of Invasive Fungal Infections. 2011;5:73–81.

[28] Warnecke L, Turner JM, Bollinger TK, Lorch JM, Misra V, Cryan PM, Inoculation of bats with European *Geomyces destructans* supports the novel pathogen hypothesis for the origin of white-nose syndrome. Proc Natl Acad Sci U S A. 2012;109:6999–7003. 10.1073/pnas.1200374109 22493237 PMC3344949

[29] de Castro F, Bolker B. Mechanisms of disease-induced extinction. Ecol Lett. 2005;8:117–26. 10.1111/j.1461-0248.2004.00693.x

[30] Mitchell KM, Churcher TS, Garner TWJ, Fisher MC. Persistence of the emerging pathogen *Batrachochytrium dendrobatidis* outside the amphibian host greatly increases the probability of host extinction. Proc Biol Sci. 2008;275:329–34. 10.1098/rspb.2007.1356 18048287 PMC2593721

[31] Casadevall A. Fungal virulence, vertebrate endothermy, and dinosaur extinction: is there a connection? Fungal Genet Biol. 2005;42:98–106. 10.1016/j.fgb.2004.11.008 15670708

[32] Powell MJ. Looking at mycology with a Janus face: a glimpse at Chytridiomycetes active in the environment. Mycologia. 1993;85:1–20. 10.2307/3760471

[33] Di Rosa I, Simoncelli F, Fagotti A, Pascolini R. The proximate cause of frog declines? Nature. 2007;447:E4–5. 10.1038/nature05941 17538572

[34] Chaturvedi V, Springer DJ, Behr MJ, Ramani R, Li X, Peck MK, Morphological and molecular characterizations of psychrophilic fungus *Geomyces destructans* from New York bats with white nose syndrome (WNS). PLoS ONE. 2010;5:e10783. 10.1371/journal.pone.0010783 20520731 PMC2875398

[35] Todd BD. Parasites lost? An overlooked hypothesis for the evolution of alternative reproductive strategies in amphibians. Am Nat. 2007;170:793–9. 10.1086/521958 17926300

[36] Ramsey JP, Reinert LK, Harper LK, Woodhams DC, Rollins-Smith LA. Immune defenses against *Batrachochytrium dendrobatidis*, a fungus linked to global amphibian declines, in the South African clawed frog, *Xenopus laevis.* Infect Immun. 2010;78:3981–92. 10.1128/IAI.00402-10 20584973 PMC2937463

[37] Joneson S, Stajich JE, Shiu S-H, Rosenblum EB. Genomic transition to pathogenicity in chytrid fungi. PLoS Pathog. 2011;7:e1002338. 10.1371/journal.ppat.1002338 22072962 PMC3207900

[38] Clark RW, Marchand MN, Clifford BJ, Stechert R, Stephens S. Decline of an isolated timber rattlesnake (*Crotalus horridus*) population: interactions between climate change, disease, and loss of genetic diversity. Biol Conserv. 2011;144:886–91. 10.1016/j.biocon.2010.12.001

[39] Robert VA, Casadevall A. Vertebrate endothermy restricts most fungi as potential pathogens. J Infect Dis. 2009;200:1623–6. 10.1086/644642 19827944

[40] Garcia-Solache MA, Casadevall A. Global warming will bring new fungal diseases for mammals. MBio. 2010;1:e00061–10. 10.1128/mBio.00061-10 PMC2912667 20689745

